# Neurocognition and Social Cognition Predicting 1-Year Outcomes in First-Episode Psychosis

**DOI:** 10.3389/fpsyt.2020.603933

**Published:** 2020-12-04

**Authors:** Maija Lindgren, Minna Holm, Tuula Kieseppä, Jaana Suvisaari

**Affiliations:** ^1^Mental Health Unit, Finnish Institute for Health and Welfare, Helsinki, Finland; ^2^Department of Psychiatry, University of Helsinki and Helsinki University Hospital, Helsinki, Finland

**Keywords:** cognition, follow-up, neuropsychology, psychotic disorders, remission

## Abstract

Cognitive performance at illness onset may predict outcomes in first-episode psychosis (FEP), and the change in cognition may associate with clinical changes. Cognitive testing was administered to 54 FEP participants 2 months after entering treatment and to 39 participants after 1 year. We investigated whether baseline cognition predicted 1-year outcomes beyond positive, negative, and affective symptoms and whether the trajectory of cognition associated with clinical change. Baseline overall neurocognitive performance predicted the 1-year social and occupational level, occupational status, and maintaining of life goals. The domain of processing speed associated with the 1-year remission, occupational status, and maintaining of life goals. Baseline social cognition associated with occupational status a year later and the need for hospital treatment during the 1st year after FEP. Most of the associations were retained beyond baseline positive and affective symptom levels, but when accounting for negative symptoms, cognition no longer predicted 1-year outcomes, highlighting how negative symptoms overlap with cognition. The trajectory of neurocognitive performance over the year did not associate with changes in symptoms or functioning. Cognitive testing at the beginning of treatment provided information on the 1-year outcome in FEP beyond positive and affective symptom levels. In particular, the domains of processing speed and social cognition could be targets for interventions that aim to improve the outcome after FEP.

## Introduction

Broad deficits in cognitive performance can be seen in first-episode psychosis (FEP), the largest deficits presenting in immediate verbal memory, executive function, and processing speed (1, 2) with social cognition being among the impaired domains (3, 4). More severe neurocognitive deficits at psychotic illness onset are linked with a more severe clinical picture and a worse prognosis, indicated by functional level, remission, and response to treatment (5, 6). In a 5-year follow-up study, higher cognitive performance at study entry predicted several domains of outcome: full recovery, functional level, and symptom remission (7). Functional outcome may also be strongly affected by social cognition (8). However, most research has been made among patients with schizophrenia instead of among the broader group of early psychosis, and methodological variability (such as the choice of participants, the cognitive predictors used, and the definition of outcomes) limits the conclusions that can be made about the association between cognition at illness onset and later functional outcomes in FEP (9).

After the acute phase of the illness, while positive symptoms may remit, cognitive deficits usually persist, affecting the daily life of the patient and possibly being more strongly predictive of functional outcome than symptomatology (9). There usually seems to be no further decline in cognitive performance after the illness onset (10). However, a subgroup of patients deteriorate further (11), and the trajectory of cognitive deficits seems to be associated with the course of psychopathology (12), specifically with symptomatic changes (13) and early relapses (14, 15). According to a meta-analysis, specific symptom changes may be related to changes in specific cognitive domains in FEP (10).

As for the clinical correlates of psychosis, cognitive impairment especially associates with negative symptoms, both also being linked with functional outcomes (16). Among recent-onset schizophrenia patients, the entry level of negative symptoms predicted social and role functioning a year later (17). It has been discussed whether negative symptoms could actually be the consequences of cognitive impairments (18, 19). In one study predicting functional outcome, there was an overlap between cognitive performance and negative symptoms so that when negative symptoms were accounted for, the psychosocial outcome was no longer predicted by baseline cognitive testing, apart from tests for processing speed and attention that had less shared variance with negative symptoms (20). Negative symptoms may also mediate or moderate the impact of cognition on function (5, 21).

Affective symptoms in psychotic disorders may also be linked to poorer cognition (22–24), but in some studies, the direction of the association has also been the reverse (25). We have previously found higher affective symptoms to associate with better cognitive performance right after getting ill (26) and with a better functional 1-year outcome in FEP (27), suggesting that anxiety and depression do not necessarily signal a poor prognosis in FEP.

Identifying the individuals with FEP at risk of less favorable outcomes may have implications for interventions. In this study, we wanted to investigate (a) whether cognitive performance at the time of entering treatment predicts 1-year clinical and functional outcomes and (b) whether the trajectory of cognitive performance during the 1st year associates with the clinical course. We took into account all the main dimensions of the psychotic illness: cognitive deficits and positive, negative, and affective symptomatology. Adding symptom dimensions to the models in stages, we wanted to investigate whether cognitive testing at baseline provides information on the outcomes independently of baseline clinical symptoms.

## Methods

### Participants

The FEP group consisted of adults making their first psychiatric treatment contact for psychosis, recruited to the Helsinki Early Psychosis Study from hospitals and outpatient clinics of the city of Helsinki and Helsinki University Hospital. They were interviewed with the Brief Psychiatric Rating Scale (BPRS), Expanded Version 4.0 (28), as soon as possible after they had commenced treatment and were able to provide informed consent, as judged by the treating personnel. As an inclusion criterion, psychosis was defined as a score ≥4 (moderate or higher) for unusual thought content or hallucinations. Exclusion criteria were psychotic disorders that were substance induced or caused by a general medical condition. The study protocol included follow-ups after 2 and 12 months using both the BPRS and the Structured Clinical Interview for the DSM-IV (SCID), Research Version (29). We utilized data from the 2-month assessment (which is when the cognitive testing was done) in order to avoid testing in the most acute phase of the illness (this is referred to here as *baseline assessment*) and from the 1-year follow-up, the time interval between the assessments being 10 months. During the follow-up period, the participants received standard treatment.

The participants gave written informed consent to participate. The study protocol was approved by the Ethics Committee of the Hospital District of Helsinki and Uusimaa and by the institutional review boards of the University of Helsinki and the Finnish Institute for Health and Welfare. The study was carried out in accordance with the sixth version of the Declaration of Helsinki.

### Cognitive Testing

Cognitive testing was administered by a psychologist at both time points: baseline and after 1 year. It included measures from the Wechsler Adult Intelligence Scale (Block Design, Vocabulary, Digit Symbol) (30), the Wechsler Memory Scale (Logical Memory, Letter-Number Sequencing, Spatial Span, Word List, Visual Reproduction) (31), the Trail Making Test (32), Verbal Fluency (33), the Tapping Task, and the Continuous Performance Test, Identical Pairs (34).

To summarize neurocognitive performance, we have previously constructed separate one-dimensional factor models for baseline (35) and after 1 year (26) (see Supplementary Table A) with a larger participant group. Factor scores for these composite factors were used as general neurocognitive indexes. Change in the composite factor over the year (the difference between the two composite factors) was used to measure the trajectory of overall neurocognitive performance.

To be able to investigate specific domains of cognitive functioning, a three-dimensional exploratory factor model of the baseline neurocognitive variables was formed and the factors were interpreted as verbal memory, speed of processing (also capturing executive functioning), and motor performance (see Supplementary Tables B1–B3).

In addition to the neurocognitive factors, the Hinting Task (36) was administered at baseline to measure the “theory of mind” domain of social cognition. We have previously found the internal consistency of the Hinting Task to be modest and obtained a one-dimensional factor solution [see Supplementary Material; (35)]. The factor scores were used in the analyses instead of the sum score of the task as they take into account the varying difficulty level and relevance of the task items. The factor is here referred to as *the social cognition factor*.

### Other Measures

Clinical assessments were conducted at baseline and 1-year follow-up. Trained research staff (nurses or psychologists) conducted the BPRS and SCID interviews. Positive psychotic symptoms were calculated as the sum of BPRS item scores for current hallucinations, unusual thought content, bizarre behavior, and conceptual disorganization. Negative symptoms were calculated as the sum of the BPRS item score for blunted affect and the alogia, anhedonia, and avolition-apathy scales of the Scale for the Assessment of Negative Symptoms (SANS) (37). The affective symptom dimension was calculated as the sum of BPRS item scores for depression and anxiety symptoms. All the baseline symptom scores used here were from the 2-month assessment (i.e., from the same time as the cognitive testing). For descriptive purposes, the use of antipsychotic medication is reported with the chlorpromazine equivalent using the DDD method (38).

Of the five outcomes used in this study, symptomatic remission after 1 year (*yes/no*) was defined—according to the criteria provided by Andreasen et al. (39)—as item scores below four (*mild*) for the symptoms of delusions, hallucinations, conceptual disorganization, blunted affect, and mannerism and posturing, as well as simultaneous scores below three for the three SANS symptoms at the time of the 1-year interview.

The level of social and occupational functioning was assessed with the Social and Occupational Functioning Scale (SOFAS) (40) on a scale of 0–100 in each study phase, and the SOFAS score after 1 year was used as a functional outcome measure.

The maintenance of a grip on life and goals in life (41) was used after 1 year to assess whether the individual had maintained age-appropriate active life goals or had given up on psychosocial goals for the future, irrespective of psychotic symptoms. Based on all the available information, it was classified as 1 = *good*, 2 = *mainly retained*, 3 = *considerably lost*, or 4 = *totally abandoned* and used as a continuous variable.

Occupational status after 1 year was based on a self-report and case records, and was defined as working (full-time or part-time work) or studying and not being on sick leave (*yes/no*) at the time of the 1-year interview.

Hospital treatment during the follow-up until the 1-year interview (*yes/no*) was coded based on self-report and case records.

### Analyses

The analyses employed IBM SPSS Statistics for Windows, version 26. The cognitive variables used as independent variables were the factor scores of the three baseline cognitive domain factors, baseline social cognition factor, baseline composite factor score, 1-year composite factor score, and the change in composite factor score over the year. 1-year outcomes of interest were remission status, the level of social and occupational functioning (the SOFAS score), maintaining life goals (grip-on-life evaluation), occupational status, and hospital treatment.

We first used Spearman correlations to examine the associations between cognition and the continuous outcome variables or the Mann-Whitney *U*-test to compare cognition according to binary outcomes. Outcomes significantly (*p* < 0.05) associating with cognition were next predicted with logistic or linear regression models. Cognitive performance was first used as the sole predictor in the first block, and in the second block, gender, age, and education level were added to the model. In the following consecutive blocks (three to five), we added baseline positive, affective, and negative symptom levels as predictors one at a time. In this way, we wanted to see whether baseline cognitive functioning added to the prediction value beyond the symptoms. Negative symptoms were added last because, based on previous literature, we expected them to overlap with cognition the most. Of the logistic models, we report the odds ratio (OR) and Nagelkerke *R*^2^ values. Of the linear models, unstandardized beta, *R*^2^, and adjusted *R*^2^ values are reported.

## Results

### Baseline Cognitive Performance and 1-year Outcomes

Baseline cognitive testing data were available from 67 FEP patients and 1-year clinical data from 54 FEP patients (Table 1), while 1-year cognitive data were available from 39 individuals. Slightly higher overall cognitive performance was found in females (composite factor: *p* = 0.040) while age was not correlated with cognition. Correlations between cognitive test performance and continuous clinical variables can be seen in Supplementary Table C. Cognitive functions were correlated inversely with negative symptoms but not with positive or affective symptoms.

**Table 1:** FEP participants with baseline cognitive data and 1-year clinical data available (*n* = 54).

**Baseline**	
Age	26.7 (5.5), 18.4–41.3
Female	24 (44.4%)
Education, years	14.5 (3.4), 9.5–23.5
SOFAS	47.9 (12.3), 25–80
Inpatient^a^	30 (55.6%)
Using antipsychotics^b^	49/53 (92.5%)
CPZE	353.0 (236.7), 0–900
Diagnosis group:^c^	
schizophrenia spectrum	32 (59.3%)
affective psychosis	12 (22.2%)
other psychotic disorder	10 (18.5%)
Cognitive performance:	
Verbal memory factor	−0.3 (1.0), −2.2–1.9
Speed of processing factor	−0.5 (0.8), −2.5–1.6
Motor performance factor	−0.3 (0.9), −2.7–2.0
Social cognition factor	−0.6 (1.7), −4.9–1.9
Composite factor	−0.4 (0.9), −2.4–1.6
**1-year**	
SOFAS	52.9 (16.2), 30–90
Remission	28/53 (51.9%)
Working or studying	26/53 (49.1%)
Hospital treatment during follow-up	9/54 (16.7%)
Grip on life, maintaining life goals	2.0 (0.8), 1–4
Using antipsychotics^d^	42/54 (77.8%)
CPZE	254.0 (225.7), 0–780
Composite factor^e^	−0.3 (1.0), −2.4–1.9

*Frequency (percent) or mean (SD) and range*.

a *Information on voluntary or involuntary treatment not reliably available*.

b *Olanzapine (28%), quetiapine (27%), risperidone (22%), and clozapine (3%)*.

c *Diagnoses were set based on all available information by a senior psychiatrist. Medical records from mental health treatment were used to complement information on symptoms provided by the SCID interview. Schizophrenia spectrum diagnoses include schizophrenia and schizophreniform disorder, and affective psychosis includes schizoaffective disorder, bipolar I disorder, and major depressive disorder with psychotic features*.

d *Olanzapine (20%), quetiapine (15%), risperidone (13%), and clozapine (11%)*.

e *n = 39*.

*CPZE, chlorpromazine equivalent; SOFAS, Social and Occupational Functioning Scale*.

#### Remission

**Figure 1:**
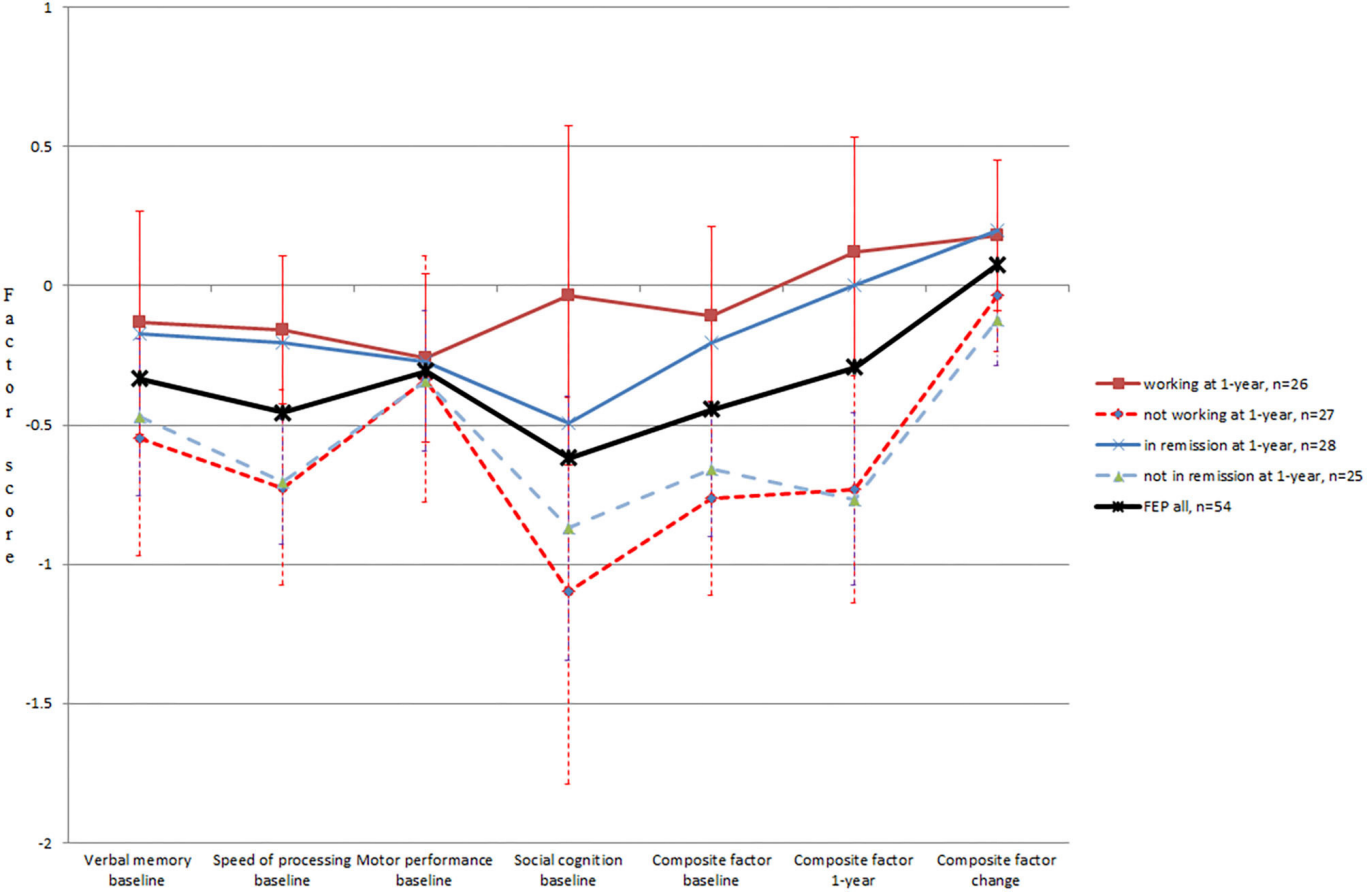
The factor scores for cognitive performance in FEP patients, based on occupational and remission status after 1 year. 1-year cognitive data available from 39 participants.

We then investigated whether baseline cognition associated with the 1-year outcomes (see Table 2). Baseline processing speed was higher among those FEP patients who were in remission after 1 year compared with those who were not, while the other baseline cognitive variables did not associate with remission. In Figure 1, cognitive performance is presented, dividing the participants based on remission status. In logistic regression models, the speed of processing continued to predict remission when controlling for education, gender, age, and baseline positive and affective symptom levels (*OR* = 2.6, *p* = 0.037; Supplementary Table D). When negative symptoms were added to the model—improving the explained variance by 38% compared to the previous block (change in *R*^2^)—the *OR* of cognition weakened from 2.6 to 1.6, losing statistical significance.

**Table 2:** Univariate associations between the five 1-year outcomes and cognitive variables.

	**Baseline**					**Change in composite factor (trajectory of neurocognition)**
	**Verbal memory factor**	**Speed of processing factor**	**Motor performance factor**	**Social cognition**	**Composite factor**	
Spearman correlations with continuous outcome measures						
SOFAS	*r* = 0.27, *p* = 0.119	*r* = 0.32,*p* = 0.052	*r* = −0.04, *p* = 0.803	*r* = 0.26,*p* = 0.114	***r*** **=** **0.34**, ***p*** **=** **0.039**	*r* = 0.32,*p* = 0.109
Grip on life^a^	*r* = −0.28, *p* = 0.064	***r*** **=** **−0.45**,***p*** **=** **0.001**	*r* = −0.03, *p* = 0.834	*r* = −0.28,*p* = 0.055	***r*** **=** **−0.43**, ***p*** **=** **0.002**	***r*** **=** **−0.33**,***p*** **=** **0.048**
Mann-Whitney tests with dichotomous outcome measures						
Remission	*U* = 337.0, *p* = 0.266	***U*** **=** **464.0**,***p*** **=** **0.042**	*U* = 333.5, *p* = 0.858	*U* = 378.0,*p* = 0.440	*U* = 456.0, *p* = 0.059	*U* = 245.0,*p* = 0.062
Working or studying	*U* = 372.0, *p* = 0.083	***U*** **=** **489.0**,***p*** **=** **0.014**	*U* = 319.5, *p* = 0.917	***U*** **=** **463.5**,***p*** **=** **0.021**	***U*** **=** **505.0**, ***p*** **=** **0.006**	*U* = 234.0,*p* = 0.224
Hospital care	*U* = 128.0, *p* = 0.344	*U* = 128.0,*p* = 0.084	*U* = 121.0, *p* = 0.170	***U*** **=** **101.0**,***p*** **=** **0.050**	*U* = 137.0, *p* = 0.128	*U* = 20.0,*p* = 0.078

*Spearman correlations or the Mann-Whitney U-test, followed with the p-value. Significant associations (p < 0.05) are in boldface*.

a *Higher values indicate the worse maintenance of life goals*.

*SOFAS, Social and Occupational Functioning Scale*.

#### Functional Outcome (SOFAS) and Maintaining Life Goals

The baseline composite factor was correlated with social and occupational functional outcome (1-year SOFAS values) (see Table 2). In linear regression models, the association was significant when positive symptoms were included (*B* = 8.3, *p* = 0.038). When affective symptoms were added to the model, cognition no longer quite reached statistical significance as a predictor (*p* = 0.054), and adding negative symptoms further diminished the significance of cognition (see Supplementary Table E).

Maintaining life goals after 1 year was assessed to be *good* or *mainly retained* in 71% of the participants. Those evaluated to have best retained their active future goals after 1 year had higher cognition when the illness had started (see Table 2). In linear regression models, the baseline composite factor predicted maintaining life goals when accounting for education, gender, age, and baseline positive and affective symptom levels (*B* = −0.45, *p* = 0.003; see Supplementary Table F). However, adding negative symptoms to the model weakened the beta of the composite factor by 76% to −0.11 while the explained variance improved by 81%. Similarly, the association between processing speed and maintaining life goals was retained in regression models controlling for all the symptom dimensions other than negative symptoms, with comparable model parameters to those of the general cognitive factor (see Supplementary Table F).

#### Occupational Status and Hospital Treatments

Working or studying at the time of the 1-year interview associated with higher cognitive performance at baseline (see Table 2, Figure 1). In regression models, occupational outcome was predicted by the baseline composite factor when controlling for education, gender, age, baseline positive symptom level, and baseline affective symptom level (*OR* = 3.0, *p* = 0.014), but not when negative symptoms were added (see Supplementary Table G). The predictors in the last block explained the variance with an *R*^2^ of 0.44 with the negative symptoms dimension increasing it by 63%. Similarly, the speed of processing domain (*OR* = 2.6, *p* = 0.035) and social cognition (*OR* = 1.8, *p* = 0.023) predicted occupational status when controlling for all the symptom dimensions other than negative symptoms (see Supplementary Table G).

Of the cognitive variables, only social cognition significantly differed between those needing and not needing hospital care during follow-up (see Table 2). However, this association was only trend-level significant after controlling for the sociodemographic variables (*OR* = 0.56, *p* = 0.054) and not significant after controlling for symptoms (see Supplementary Table H).

### The Trajectory of Cognitive Performance and Clinical Course

The 1-year composite factor was correlated with the baseline composite factor (*r* = 0.81, *p* < 0.001), and change in the composite factor during the 1st year after FEP was minor on average (0.08 ± 0.51; see Figure 1). Males more often improved their performance over the year (*p* = 0.003). Improved cognitive level also was correlated with milder baseline and 1-year positive symptoms, and 1-year affective symptoms, as well as better maintaining life goals, but it was not correlated with the other outcomes. Change in cognition was not linked to change in positive, negative, or affective symptom levels or functioning score during the year (see Table 2, Supplementary Table C). During the follow-up period, all symptom levels improved: positive symptoms from 3.2 ± 3.9 to 1.6 ± 2.4, negative symptoms from 5.3 ± 4.0 to 4.9 ± 4.3, and affective symptoms from 5.3 ± 2.9 to 4.3 ± 2.5.

## Discussion

In this study, cognitive functioning at the beginning of treatment was used to predict outcomes after 1 year in individuals with a recent FEP. We assessed both the clinical outcome (remission, hospital treatment) and the functional outcome (the SOFAS level, occupational status, maintaining life goals). Cognitive factors predicted the outcomes and most of the associations were retained beyond baseline positive and affective symptom levels but not beyond negative symptoms.

A higher composite factor score for cognition predicted working or studying after 1 year and higher evaluations for both maintaining life goals and the level of social and occupational functioning (evaluated using SOFAS) after 1 year. As specific cognitive domains may be predictive of specific functional outcomes (8), we also separated neurocognition into three domains. Of the domains, higher processing speed predicted a better 1-year outcome in terms of remission, occupational status, and maintaining life goals, whereas the verbal memory and motor performance factors did not show statistically significant associations with the 1-year clinical or functional outcomes. Tests loading most on the speed factor were the Trail Making test, Digit Symbol, and Verbal Fluency (see Supplementary Table B1). Although processing speed is among the cognitive domains with the largest deficits (1), few studies have found it to specifically associate with outcomes, while verbal memory often seems to be most related to clinical outcome (42). For example, verbal memory impairment predicted sustained remission in the early stage of schizophrenia (43). The associations between our verbal learning and memory factor and the outcomes were in the expected direction, although not significant. It should also be noted that the high loadings on verbal learning on our composite cognitive factor (see Supplementary Table A) predicting several outcomes was in line with these earlier studies.

Impaired social cognition predicted occupational status and associated with the need for hospital treatment, although this association was not retained in multivariate analyses when accounting for all the background and clinical factors. Deficits in the processing of social information, such as maladaptive interpretations and reactions in social situations, strongly affect everyday functioning (44). In schizophrenia, studies have shown even larger effects of social cognition on functional outcome than neurocognition (8, 45). Social cognition may also affect the relationship between neurocognition and functional level (3, 46) so that neurocognitive deficits have more effect if social cognitive abilities are also impaired. In a multicenter study among people with schizophrenia, neurocognition strongly predicted functioning but did so indirectly, one of the mediators being social cognition (47). Although this mechanism was not investigated here, it should be noted that general neurocognition and processing speed associated with the outcomes more often than social cognition.

Previous literature shows that cognitive assessment conducted at treatment entry may predict the later functional outcome (48) and cognitive deficits at illness onset can thus be seen as prognostic markers (9). For example, in one FEP study, over half of the variance in occupational outcome at 9 months was explained by baseline neurocognitive factors (49), which is a stronger effect than in the current study. In line with our results, the previous studies have found larger relationships with global measures of cognition, but associations have also been found concerning the domains of verbal memory and executive functioning, among others (50). According to a review among patients with schizophrenia, cognitive deficits predict trajectories in many everyday functional domains, such as work or school ability, social relationships, and self-care (5). Another review on FEP found that remission within the first 2 years of illness was best predicted by verbal fluency, memory, and social cognition and that functional outcomes were best predicted by verbal memory (6). In the current study, we found that similar types of outcomes were predicted by baseline cognition, but with an emphasis on different cognitive domains compared with Schubert's review.

Inconsistency in previous findings may be explained by the cognitive assessments administered and the cognitive domains formed, varying lengths of follow-up, and the definition of the outcomes (9, 42). In our study, *remission* was defined as a symptom level of mild or lower in regard to positive, negative, and disorganized symptoms at the time of a 1-year assessment, while some other studies demand a period of decreased symptoms, for example, 6 months. In addition to remission, we used four other outcome measures to reflect clinical and functional recovery from the first episode. In the context of recovery orientation (51), pursuing or achieving personal life goals can be seen as more relevant than the mere absence of symptoms. That is why one of our outcomes of interest was the maintenance of life goals. The life goals of individuals hospitalized for FEP typically include achieving employment or education, building or strengthening social relationships, having independent housing, and having better physical or mental health (52). In the current study, maintaining life goals after 1 year associated with higher cognition at illness onset.

In our analyses, we controlled for positive, negative, and affective symptoms and one of the main results was that cognition did not predict 1-year outcomes when taking into account the effect of negative symptoms. It has been noted that although cognitive processes predict the functional outcome, a large proportion of the variance remains to be explained by other factors, such as negative symptoms (8). Previous literature shows that poor pre-morbid adjustment, severe negative symptoms, and male gender are associated with a worse outcome in FEP (53). The relationship between negative and cognitive symptoms seems complex: They have been suggested to share etiology (54) or have a causal relationship (18). They may be manifestations of the same phenomenon and have synergistic impact on functioning: a person with severe negative symptoms may not show high effort and motivation in a neuropsychological testing situation, or cognitive functioning could affect the manifestation and assessment of negative symptoms. The question of how separable or overlapping cognition and negative symptoms are affects the possibility that interventions targeting one could also affect the other. In one FEP study, the benefits of cognitive remediation extended to include negative symptom reduction and improved social functioning (55). On the other hand, cognitive and negative symptoms may have different effects on outcomes, so cognition would associate with the *ability* for everyday functioning and negative symptoms with the *likelihood* of everyday functioning (16). Negative symptoms and cognitive deficits may also predict different outcomes in schizophrenia, negative symptoms predicting social functioning and cognition predicting occupational functioning and the capacity to carry out everyday activities (56). This influences the effect that cognitive remediation has on different aspects of disability.

Further, the definition and measurement of negative symptoms affects their overlap with cognitive abilities (57). Subgroups with distinct negative symptom profiles can be identified among schizophrenia patients, showing differences in cognition as well as in functional and clinical outcomes (58, 59). Of the domains of negative symptoms, avolition may have larger associations with functioning than poor emotional expression (47). All in all, the boundaries between negative symptoms and cognition are not well-defined (60). Negative symptoms may also affect the relationship between cognition and outcome (5, 21). A recent prospective psychosis study showed that at least some of the cognitive impairments were driven by negative symptoms, cognitive performance mediating the relationship between negative symptoms and long-term outcome (61). This led to a suggestion that by treating negative symptoms, progressive decline could be prevented (61).

In the current study, negative symptoms shared variance with the cognitive performance so that including negative symptoms in the models predicting the outcomes decreased the impact of cognition to a non-significant level. Negative symptoms especially predicted maintaining life goals, the SOFAS level, and occupational status. Similarly, in some previous studies predicting functional outcomes in schizophrenia, cognitive testing often did not add predictive value when also accounting for negative symptoms (20, 62). In a 2-year follow-up study among first-episode schizophrenia-spectrum disorders, symptomatic and functional remission, and quality of life were all associated with higher cognitive performance but associations did not remain significant in regression models controlling for symptoms (63).

In our study, processing speed could be seen to specifically associate with the outcomes, again overlapping with baseline negative symptoms. As for factors combining processing speed and negative symptoms, antipsychotic medication may slow down cognitive processes and increase the manifestation of negative symptoms. Also, extrapyramidal symptoms and other motor impairments could have a role in the relationship between cognition and negative symptoms, both as the side effects of antipsychotics and as primary traits in psychotic disorders (64, 65). Both a toxic effect of antipsychotic treatment on cognition (66) and a protective effect on it (48, 67) have been suggested, but cognitive deficits are also evident in drug-naïve schizophrenia patients (68). Higher antipsychotic doses are often a consequence of more severe symptoms impairing cognition (69) which complicates investigating the issue. An optimal level of medication is difficult to set out, and lower or higher doses cannot be determined to be better outcomes. Also, the place of treatment should be taken into account; on entering a hospital, the dosage of antipsychotic medication may be higher, whereas in outpatient care, a good outcome could associate with a higher level of medication rather than with a level that is too low. For these reasons, we did not control for antipsychotic or other types of medication (SSRI medication was used at 2 months by 21% and at 12 months by 26% of the participants), however, medication used can affect cognitive testing and performance. We also did not control for alcohol or substance use; of note, cannabis had been used by 42%, current alcohol use disorder was diagnosed in 5%, and current substance use disorder in 2% of the participants at baseline. Although the participants did not appear to be under the influence of substances or alcohol at the appointments, we did not formally assess this, which may have affected the results.

Contrary to negative symptoms, the role of affective and positive symptoms in predicting 1-year outcomes seemed quite small. We also found that while cognitive functions (especially those in the verbal, speed, and social cognition domains) were correlated with negative symptoms, they were not correlated with positive or affective symptom levels. Our sample included both affective and non-affective psychotic disorders, and we did not account for diagnosis in our relatively small sample. Generally, affective symptoms in FEP may associate with a more severe clinical picture (70). Our previous results suggest that affective symptoms right after getting ill may associate with better cognitive performance (26) as well as with a better functional 1-year outcome (27), which possibly relates to better insight into the situation that evokes negative emotions and a better understanding of the situation. Positive symptoms of psychosis are seldom associated with cognitive functioning (54), and they also seem to interfere with everyday functioning less than negative symptoms (21).

Changes in cognitive performance over the 1st year after FEP could not be linked to concurrent changes in symptom or functioning levels. One reason for this can be that there was little change in the composite factor over the year, in line with previous works showing relatively stable cognitive levels after the first episode (10). Change in cognition was related to baseline cognition, so those with a good baseline level had the biggest decline during the year, declining the feasibility of using the cognitive change variable. Improved neurocognition associated with milder positive symptom levels but was not associated with negative symptom levels at either time point. Previous studies have found the cognitive trajectory to predict illness severity: in a 3-year follow-up, cognitive deterioration was associated with more negative and disorganization symptoms, and with worse occupational outcomes (11). In a 10-year follow-up, remission after 1 year associated with a better neurocognitive course, especially in verbal memory (14, 15). A better clinical situation, especially a decrease in negative symptoms, can be reflected as an improvement in cognitive performance (12). Cognitive improvement also associated with symptomatic change in the study by Anda and colleagues, and changes in negative symptoms were more relevant than the baseline negative symptom level (13). In another study, outcomes in cognition, the recovery of functioning, and clinical illness progression were all related to each other in the course of the disease (69). However, compatible with our results, some other studies have not found cognitive change to associate with clinical change (16).

### Strengths and Limitations

We followed up the patients for 12 months after treatment onset, the follow-up period being 10 months between the assessment time points. Some participants were lost at follow-up, but those with or without 1-year clinical data did not differ in baseline cognition or functioning. The 1st year after treatment initiation can be seen as a critical period in the course of illness, after which the situation has often stabilized. In a 10-year follow-up study, relapses during the 1st year associated with neurocognitive trajectories, while relapses later on did not (14). The longer follow-up of the current sample will be addressed later.

Cognitive performance was assessed with composite factors formed of baseline and 1-year testing. As broader testing was done at baseline, the factor solutions were not identical; however, the tests that were included at both time points loaded on the composite factor in a similar order (see Supplementary Table A). After 1 year, only the composite factor was used and the domains of cognition were not investigated separately.

We did not assess functional capacity with performance-based measures that possibly mediate the relationship between neurocognition and functional level (47, 56).

If we had corrected for multiple testing, the associations in Table 2 concerning remission, functional outcome, and hospital treatments would have not stayed statistically significant. However, the results concerning occupational status and maintaining life goals would have remained significant, stressing the association of cognition with these everyday outcomes.

## Conclusion

Cognitive symptoms could serve as prognostic markers in FEP, predicting outcomes, making neurocognitive abilities and social cognition the key drivers of recovery. Cognitive deficits may also affect the outcome via collaboration in treatment (47). Among the most informative neuropsychological tests conducted at treatment entry were those assessing verbal learning (Word List, Logical Memory) and verbal fluency, as well as those assessing executive functioning and processing speed (Trail Making, Digit Symbol). In addition, social cognition associated with several outcomes. Our results suggest that cognitive functions highly overlap with negative symptomatology, both predicting a worse outcome. Because of the devastating effects of negative symptoms and cognitive impairment, our results also stress the possibility of cognitive remediation and social cognitive training helping those with FEP. Interventions targeting early cognitive impairments could affect the course of the illness.

## Data Availability Statement

The data analyzed in this study is subject to the following licenses/restrictions: The data that has been used is confidential. Data are from the Helsinki Early Psychosis Study at the Finnish Institute for Health and Welfare. Sharing of the data is possible in research collaborations if it is in agreement with the consent given by the participants and with the General Data Protection Regulation (GDPR) and other applicable law. Collaborations require a separate agreement and local ethical committee approval. Requests to access these datasets should be directed to Jaana Suvisaari, jaana.suvisaari@thl.fi.

## Ethics Statement

The studies involving human participants were reviewed and approved by Ethics Committee of the Hospital District of Helsinki and Uusimaa; Institutional review boards of the University of Helsinki and the Finnish Institute for Health and Welfare. The patients/participants provided their written informed consent to participate in this study.

## Author Contributions

JS and TK were principal investigators in the Helsinki Early Psychosis Study and were designing the original study protocol. ML and MH participated in collecting the data, and JS was responsible for DSM-IV diagnoses. ML undertook the statistical analysis and wrote the first draft of the manuscript. All authors contributed to and have approved the final manuscript.

## Conflict of Interest

The authors declare that the research was conducted in the absence of any commercial or financial relationships that could be construed as a potential conflict of interest.
