# Supplementary material for: Neurocognition and Social Cognition Predicting 1-Year Outcomes in First-Episode Psychosis
Source: Front Psychiatry. 2020 Dec 4;11:603933. doi: 10.3389/fpsyt.2020.603933 (PMC7746550; doi:10.3389/fpsyt.2020.603933)
Supplement: Supplementary file 1 [file Data_Sheet_1.PDF]

Supplement: Neurocognition and social cognition predicting one-year outcomes in first-episode psychosis

Supplementary table A. Factor loadings in the baseline and one-year composite factors

	Factor loading	
Task variable	Baseline composite factor	One-year composite factor
WMS-III Word List, immediate	.82	-
WMS-III Logical Memory, delayed	.77	.78
Trail Making Test B [a]	.76	.76
WAIS-III Digit Symbol	.75	.75
WMS-III Logical Memory, immediate	.73	.74
Trail Making Test C, alphabets [a]	.73	-
Verbal Fluency	.70	.70
WMS-III Word List, long delay	.69	-
WMS-III Letter-Number Sequencing	.64	.64
WAIS-III Block Design	.59	.59
WAIS-III Vocabulary	.56	.57
Trail Making Test A [a]	.56	.56
WMS-III Logical Memory, delayed recognition	.52	.53
WMS-III Visual Reproduction, delayed	.50	-
CPT-IP mean	.50	.50
WMS-III Word List, delayed recognition	.49	-
WMS-III Spatial Span	.45	.45
WMS-III Visual Reproduction, immediate	.33	-
Tapping, nondominant hand	.26	.26
Tapping, dominant hand	.26	.26

- test not done at one-year

[a] Trail Making Tests inverted

CPT-IP, Continuous Performance Test - Identical Pairs

A single-dimensional factor model of the neurocognitive variables was formed with Mplus using MLR estimation to summarize cognitive performance separately for baseline and one-year testing data. *The maximum a posteriori* factor scores for this composite factor were used as an overall index of general neurocognitive performance.

In addition, dichotomized Hinting Task data were analyzed with Mplus to obtain a single-dimensional WLSMV factor solution. Factor scores were estimated with the *maximum a posteriori* method. Factor scores were normalized based on the control group distribution.

References:

Lindgren, M., Birling, H., Kieseppä, T., Tuulio-Henriksson, A., 2020. Is cognitive performance associated with anxiety and depression in first-episode psychosis? J. Affect. Disord. 263, 221–227.

Lindgren, M., Torniainen-Holm, M., Heiskanen, I., Voutilainen, G., Pulkkinen, U., Mehtälä, T., Jokela, M., Kieseppä, T., Suvisaari, J., Therman, S., 2018. Theory of mind in a first-episode psychosis population using the Hinting Task. Psychiatry Res. 263, 185–192.

Supplementary table B1. Standardized loadings on the three exploratory neurocognitive domain factors.

Test variable	F1 Verbal memory	F2 Speed of processing	F3 Motor performance
WMS-III Logical Memory, delayed	0.99	0.01	0.00
WMS-III Logical Memory, immediate	0.94	0.03	0.02
WMS-III Word List, delayed recognition	0.81	0.19	0.06
WMS-III Logical Memory, delayed recognition	0.73	0.08	0.14
WMS-III Word List, long delay	0.69	0.10	0.12
WMS-III Word List, immediate	0.63	0.31	0.02
Trail Making Test A [a]	0.22	0.86	0.04
Trail Making Test C, alphabets [a]	0.01	0.80	0.05
WAIS-III Digit Symbol	0.12	0.79	0.09
Trail Making Test B [a]	0.15	0.71	0.03
Verbal Fluency	0.15	0.69	0.04
WAIS-III Block Design	0.06	0.55	0.22
WMS-III Spatial Span	0.14	0.52	0.26
WMS-III Letter-Number Sequencing	0.26	0.45	0.16
CPT-IP mean	0.02	0.45	0.28
WAIS-III Vocabulary	0.23	0.41	0.13
Tapping Task, dominant hand	0.13	0.02	0.86
Tapping Task, nondominant hand	0.04	0.03	0.83
WMS-III Visual Reproduction, immediate	0.31	0.07	0.55
WMS-III Visual Reproduction, delayed	0.29	0.18	0.51

[a] Trail Making Tests inverted
CPT-IP, The Continuous Performance Test, Identical Pairs
WAIS-III, Wechsler Adult Intelligence Scale - Third Edition
WMS-III, Wechsler Memory Scale - Third Edition

Supplementary table B2. Linear correlations between the three exploratory neurocognitive domain factors.

Test variable	F1 Verbal memory	F2 Speed of processing	F3 Motor performance
F1 Verbal memory	1		
F2 Speed of processing	0.56	1	
F3 Motor performance	0.11	0.34	1

To summarize neurocognitive performance, a three-dimensional exploratory factor model of twenty-two cognitive test variables was constructed with Mplus 7.1 using maximum likelihood estimation (parameters in Supplementary tables B1 and B2). We used oblimin rotation, as the factors were assumed to correlate with each other. The model was exploratory because we wanted

to find the latent factors best fitting our data. We included all the principal variables from the tests in our test battery, which was designed to capture the significant areas of cognitive performance in psychosis. The factor model was formed with 129 participants (including control participants who were not used in the current study). The number of factors was fixed to three, based on model fit and interpretability of the factors. Factor scores were calculated with the *maximum a posteriori* method. These methods allow for the presence of missing data, which was assumed to be missing at random. The three-dimensional factor model had tolerable fit indices (Supplementary table B3), and the factors were interpretable as Verbal Learning and Memory, Speed of Processing (also covering executive functioning), and Motor Performance. Though the four-dimensional model had marginally better fit indices, the last two factors represented only the subtasks of one test each (Visual Reproduction and Tapping), and was therefore not considered an improvement, and the three-dimensional model was chosen for parsimony. The factor score determinacies for the complete-data pattern were good (.99, .95, and .93 respectively).

Supplementary table B3. Model fit for exploratory factor models.

Exploratory Model	CFI	RMSEA [90% C.I]	SRMR	ECV
2-dimensional	0.80	0.11 [0.10, 0.12]	0.08	46 %
3-dimensional	0.89	0.09 [0.07, 0.10]	0.07	53 %
4-dimensional	0.91	0.08 [0.07, 0.10]	0.05	58 %

CFI, Comparative Fit Index
RMSEA, Root mean square error of approximation
SRMR, Standardized Root Mean Square Residual
ECV, Explained Common Variance

References:

Benton, A.L., Hamsher, K., 1976. Multilingual Aphasia Examination. University of Iowa, Iowa City.

Cornblatt, B.A., Risch, N.J., Faris, G., Friedman, D., Erlenmeyer-Kimling, L., 1988. The continuous performance test, identical pairs version (CPT-IP): New findings about sustained attention in normal families. Psychiatry Res. 26, 223–238.

Reitan, R.M., Wolfson, D., 1985. The Halstead-Reitan neuropsychological test battery. Neuropsychological Press, Tucson, Arizona.

Wechsler, D., 1997a. Wechsler Adult Intelligence Scale - Third Edition. The Psychological Corporation, San Antonio, Texas.

Wechsler, D., 1997b. Wechsler Memory Scale - Third Edition. The Psychological Corporation, San Antonio, Texas.

Supplementary table C. Correlations between clinical and cognitive variables

	Baseline						
	Verbal memory factor	Speed of processing factor	Motor performance factor	Social cognition	Composite factor	One-year composite factor	Change in composite factor
Baseline SOFAS	0.38 (.003)	0.40 (.001)	-0.01	0.43 (.001)	0.42 (.001)	0.61 (<.001)	0.72
SOFAS change	-0.24	-0.08	-0.01	-0.01	-0.03	0.11	0.33
Baseline positive symptoms	0.16	-0.03	-0.04	-0.19	0.02	-0.08	-0.45 (.004)
One-year positive symptoms	0.01	-0.03	0.02	0.21	-0.05	-0.18	-0.34 (.035)
Positive symptoms change [a]	-0.25	0.02	0.10	0.35 (.012)	-0.11	-0.02	0.29
Baseline negative symptoms	-0.42 (.001)	-0.45 (<.001)	-0.12	-0.47 (<.001)	-0.50 (<.001)	-0.61 (<.001)	-0.00
One-year negative symptoms	-0.35 (.015)	-0.47 (<.001)	-0.05	-0.34 (.015)	-0.49 (<.001)	-0.70 (<.001)	-0.28
Negative symptoms change [b]	0.08	-0.03	0.20	-0.03	-0.02	-0.24	-0.32
Baseline affective symptoms	0.17	0.15	-0.25	-0.07	0.24	0.21	-0.13
One-year affective symptoms	0.09	0.09	-0.05	0.01	0.11	-0.13	-0.35 (.028)
Affective symptoms change [c]	-0.16	-0.15	0.14	-0.16	-0.24	-0.31	-0.07

SOFAS, Social and Occupational Functioning Scale
Statistically significant correlations ($p < .05$) followed with the *p*-value.

Supplementary table D. Logistic regression model predicting remission after one year. The odds ratio (*OR*) and the significance (*p*) of the Speed of processing factor and the Nagelkerke R^2 of the models.

Predictors in the model	OR (speed factor)	95% CI lower	95% CI upper	p	R²
Speed factor	2.2	1.0	4.7	.054	.12
Speed factor + age + gender + education	2.4	1.0	5.9	**.048**	.18
Speed factor + age + gender + education + positive symptoms	2.6	1.1	6.5	**.036**	.26
Speed factor + age + gender + education + positive symptoms + affective symptoms	2.6	1.1	6.5	**.037**	.26
Speed factor + age + gender + education + positive symptoms + affective symptoms + negative symptoms	1.6	.57	4.3	.385	.36

Supplementary table E. Linear regression model predicting the SOFAS score after one year. The beta coefficients (*B*) and the significance (*p*) of the Composite factor and the R^2 of the models.

Predictors in the model	B (composite factor)	95% CI lower	95% CI upper	p	R²	adjusted R²
Composite factor	7.2	.81	13.5	**.028**	.13	.11
Composite factor + age + gender + education	7.6	-1.1	16.3	.085	.13	.02
Composite factor + age + gender + education + positive symptoms	8.3	.50	16.1	**.038**	.33	.22
Composite factor + age + gender + education + positive symptoms + affective symptoms	7.9	-.14	15.9	.054	.34	.21
Composite factor + age + gender + education + positive symptoms + affective symptoms + negative symptoms	1.8	-5.6	9.2	.626	.56	.45

Supplementary table F. Linear regression model predicting maintaining life goals after one year [a]. The beta coefficients (*B*) and the significance (*p*) of the cognitive factor (Composite or Speed factor), and the R^2 of the models.

Predictors in the model	B (cognitive factor)	95% CI lower	95% CI upper	p	R^2	adjusted R^2
Composite factor						
Composite factor	-.45	-.71	-.20	**.001**	.23	.21
Composite factor + age + gender + education	-.43	-.74	-.13	**.006**	.23	.16
Composite factor + age + gender + education + positive symptoms	-.45	-.73	-.17	**.002**	.36	.28
Composite factor + age + gender + education + positive symptoms + affective symptoms	-.45	-.73	-.17	**.003**	.37	.27
Composite factor + age + gender + education + positive symptoms + affective symptoms + negative symptoms	-.11	-.36	.20	.569	.57	.49
Speed of processing factor						
Speed factor	-.44	-.72	-.17	**.002**	.19	.18
Speed factor + age + gender + education	-.40	-.71	-.09	**.013**	.20	.13
Speed factor + age + gender + education + positive symptoms	-.41	-.70	-.13	**.006**	.33	.25
Speed factor + age + gender + education + positive symptoms + affective symptoms	-.41	-.70	-.12	**.007**	.34	.24
Speed factor + age + gender + education + positive symptoms + affective symptoms + negative symptoms	-.08	-.36	.20	.569	.56	.48

[a] higher values indicate worse outcome

Supplementary table G. Logistic regression model predicting working or studying after one year. The odds ratio (*OR*) and the significance (*p*) of the cognitive factor (Composite, Speed, or Social cognition factor), and the Nagelkerke R^2 of the models.

Predictors in the model	OR (cognitive factor)	95% CI lower	95% CI upper	p	R^2
Composite factor					
Composite factor	2.7	1.2	5.8	**.015**	.18
Composite factor + age + gender + education	3.0	1.2	7.4	**.015**	.20
Composite factor + age + gender + education + positive symptoms	3.1	1.3	7.5	**.013**	.25
Composite factor + age + gender + education + positive symptoms + affective symptoms	3.0	1.3	7.4	**.014**	.27
Composite factor + age + gender + education + positive symptoms + affective symptoms + negative symptoms	1.6	.57	4.4	.385	.44
Speed of processing factor					
Speed factor	2.5	1.1	5.5	**.028**	.14
Speed factor + age + gender + education	2.5	1.0	5.9	**.039**	.15
Speed factor + age + gender + education + positive symptoms	2.6	1.1	6.1	**.032**	.20
Speed factor + age + gender + education + positive symptoms + affective symptoms	2.6	1.1	6.1	**.035**	.22
Speed factor + age + gender + education + positive symptoms + affective symptoms + negative symptoms	1.3	.46	3.7	.623	.43
Social cognition factor					
Social cognition	1.7	1.1	2.5	**.014**	.18
Social cognition + age + gender + education	1.8	1.1	2.9	**.021**	.19
Social cognition + age + gender + education + positive symptoms	1.8	1.1	2.9	**.022**	.22
Social cognition + age + gender + education + positive symptoms + affective symptoms	1.8	1.1	3.1	**.023**	.26
Social cognition + age + gender + education + positive symptoms + affective symptoms + negative symptoms	1.5	.84	2.6	.176	.47

Supplementary table H. Logistic regression model predicting hospital care during follow-up. The odds ratio (*OR*) and the significance (*p*) of the Social cognition factor, and the Nagelkerke R^2 of the models.

Predictors in the model	OR (social cognition)	95% CI lower	upper	p	R^2
Social cognition	.60	.37	.97	**.035**	.16
Social cognition + age + gender + education	.56	.32	1.0	.054	.28
Social cognition + age + gender + education + positive symptoms	.58	.33	1.0	.069	.29
Social cognition + age + gender + education + positive symptoms + affective symptoms	.55	.29	1.0	.060	.39
Social cognition + age + gender + education + positive symptoms + affective symptoms + negative symptoms	.63	.32	1.2	.180	.47
